# Bentonite-based functional material as preconcentration system for determination of chromium species in water by flow injection analysis technique

**DOI:** 10.1016/j.heliyon.2020.e04051

**Published:** 2020-05-23

**Authors:** Muhammad Bachri Amran, Sitti Aminah, Handajaya Rusli, Buchari Buchari

**Affiliations:** a Analytical Chemistry Research Group, Faculty of Mathematics and Natural Sciences, Institut Teknologi Bandung, Indonesia; b Department of Chemistry Education, Tadulako University, Palu, Indonesia

**Keywords:** Analytical chemistry, Environmental science, Physical chemistry, Preconcentration, Trace analysis, Cr species, Modified bentonite, FIA-AAS

## Abstract

Chromium species have different level of toxicities. For example, Cr(VI) is 100 times more toxic than Cr(III). This characteristic makes speciation analysis of chromium become important. This research will discuss about a development of a Flow Injection Analysis-Atomic Absorption Spectrometry (FIA-AAS) technique that utilizes bentonite based functional material as a retention column. The separation, preconcentration and trace analysis of the Cr species in the water has been successfully performed using a Bt-MCCs mini-column in the FIA-AAS system. Analytical performance of the developed method is described as repeatability, linearity, and detection limit. Analytical performance for Cr(III) are 1.78 %, correlation coefficient 0.9975 for the concentration range of 50–600 μg.L^−1^, and 2.76 μg.L^−1^, respectively. Whereas, analytical performance for Cr(VI) are 0.60 %, correlation coefficient 0.9926 for concentration range of 50–600 μg.L^−1^, and 2.42 μg.L^−1^, respectively. This limit detection is better than the other selective method that has been reported using AAS as detector and the concentration range can be widened with this limit detection. Evaluation of FIA performance for both of Cr(III) and Cr(VI)is that it has an enrichment factor of 10 times higher, it has a concentration efficiency of 12 h^-1^ and it has a consumptive index of 12 mL. The analysis that was obtained in Cidurian River, West Java, Indonesia are 38.28 g.L^−1^ for Cr(III) and 26.73 g.L^−1^ for Cr(VI), while the accuracy are 98.84 % for Cr(III), and 100.73 % for Cr(VI).

## 1 Introduction

Anthropogenic activities can harm in environment. It uses heavy metal objects as their culture and can result into water pollution when it is being thrown away to the river. The heavy metal ions can give negative impacts on health and the environment due to its high level of toxicity and a risk of spreading its toxic through the food chain of animals. Chromium (Cr) and its compounds are one of the metals that are popular in various industries such as electroplating, tanning, and paint [1, 2, 3]. Chromium is one of the trace elements that can be very useful for the human being, at the same time pose serious risks depending on their species. Cr(VI) has 100 times toxicity level higher than Cr(III). Cr(III) is essential for glucose, lipid and protein metabolism in mammals. While Cr(VI) is carcinogenic and toxic for biology system [4], so it is important to determine the concentration each of Cr [5, 6].

Many of Cr speciation using column have been developed such as, Solid Phase Extraction-AAS [7, 8, 9], Inductive Coupled Plasma-Mass Spectrometry (ICP-MS) [10, 11], Ionic Chromatography-ICP-MS [12], High Performance Liquid Chromatography (HPLC)-ICP-MS [13], and Reverse Phase-HPLC [14]. Those methods are difficult to be applied because the instrument is relatively expensive and have complicated procedure analysis. AAS is one of the standard instruments that is used for metal analysis, but its instrument cannot be used for speciation analysis and does not have a good sensitivity for trace analysis. Given the aforementioned explanation, we need separation and pre concentration before the analysis using AAS to deal with the issue.

The alternative method for preconcentration and trace analysis of metal ion is a non-chromatography mini-column technique based on FIA with AAS as detector. The advantages of FIA method compared to other conventional preconcentration method are; low cost, high repeatability, fewer sample needed, relatively short time for analysis, and easy to couple with any detection methods [15, 16]. Many strategies have been developed to find Cr speciation method based on FIA for example using a functional material as sorbent in FIA system. This sorbent can get through modification of a natural material or synthesis of new material. Chelating resin is one of the popular sorbent using in FIA system such as; C-18 column with 1,5-diphenyl carbazide (DPC) as a ligand in FIA-UV-Vis [17], functionalized alumina surfactant with 8-hidroquinoline in FIA-AAS [18], modified XAD in FIA-AAS [19, 20]. All these FIA systems have a limitation. They can only analyze one of Cr species. So, it is needed to develop an alternative sorbent which has a good performance to determine Cr(III) and Cr(VI) species using FIA system.

This research managed to develop and evaluate a new FIA-AAS technique utilizing a modified bentonite as a functional material for low cost preconcentration system with good analytical performance. Modified bentonite as sorbent has not reported yet to the best of our knowledge. The functional material was made from bentonite which was modified with cetyltrimethylammonium bromide (CTAB) and chitosan. This material could improve the weakness of AAS technique (sensitivity, selectivity and detection limit) for Cr species analysis. The combination with FIA technique can produce a new technique which are able to determine Cr species simultaneously with being selective in trace concentration. These abilities expected are expected to give a contribution for determination of Cr(III) and Cr(VI) species in the environment.

## 2 Materials and methods

### 2.1 Synthesis of bentonite functional material (Bt-MCCs)

The natural bentonite was obtained from Cipatat, West Java, Indonesia and it was used without any further treatment. Cr(NO_3_)_3_.9H_2_O and K_2_Cr_2_O_7_ were used as Cr(III) and Cr(VI) standard solution. CTAB, sodium hydroxide, nitric acid, ammonia solution and acetic acid glacial were taken from Merck-Germany. While chitosan DD 81 % was from Biotech. All chemicals used in this research were pro analyst grade except mentioned differently. The Bt-MCCs material has been synthesis with a previous procedure that was reported by Aminah [21].

Five grams of natural bentonite with particle size +200/-100 mesh dispersed in 250 mL of 0.01 mol.L^−1^ CTAB solution and stirred for 24 h at room temperature. Then, the product was filtered and washed with distilled water until bromide ion became undetectable. Then, dried at 60 °C for 24 h. Afterwards, this aggregate crushed and sieved to obtain particle size +200/-100 mesh. This material is called Bt-MCs. Five grams of Bt-MCs then added to 250 mL of distilled water to get a 2.0 % suspension. On the other hand, a chitosan solution was prepared by dissolving five grams of chitosan DD 81 % in 250 mL of 1.0 % acetic acid solution. The chitosan solution added slowly to suspension and stirred for 24 h at room temperature. The formed materials were washed with distilled water until neutral pH and then dried at 60 °C for 24 h. Finally, the aggregate was crushed and sieved to obtain particle size +200/-100 to get a Bt-MCCs materials.

### 2.2 FIA-AAS system

**Figure 1:**
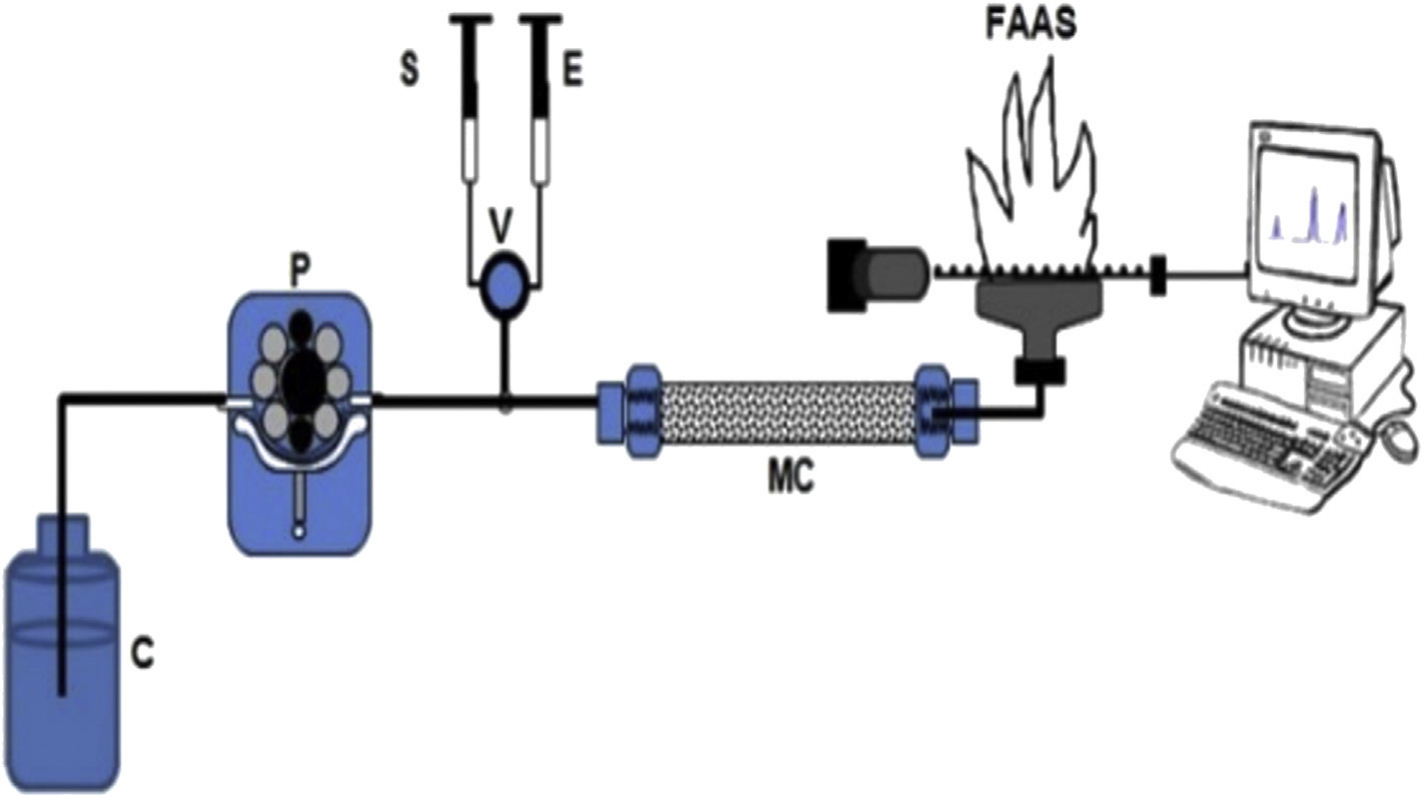
Diagram of FIA-AAS volume-based system (C: Carrier; S: Sample; E = Eluent; P = Peristaltic pump; MC: Bt-MCCs mini-column; FAAS: Flame Atomic Absorption Spectrometer).

In this system, a flow injection was operated based on volume to study eluent volume, eluent concentration, and sample volume effects. Determination of Cr(III) and Cr(VI) was done using AAS. A peristaltic pump, a valve and a loop were used to give elution pressure, elution control, and controlling of sample volume, respectively. For application, 0.2 g of Bt-MCCs material was packed in mini-column. To get the best result, optimization and evaluations of FIA system was done. The evaluation and optimization of sample volume, kind of eluent, eluent concentration, and volume of eluent were applied to get best result. The eluent for Cr(III) and Cr(VI) is nitric acid and ammonia solution, respectively. The carrier in this system was a water of pH 5, which acidified by nitric acid. The concentration of Cr(III) and Cr(VI) that was used in this experiment to study the concentration and volume eluent is 250 μg.L^−1^. The flow of the carrier was 2.0 mL min^−1^. Diagram for FIA-AAS volume-based system illustrated in Figure 1 [22].

## 3 Results

**Figure 2:**
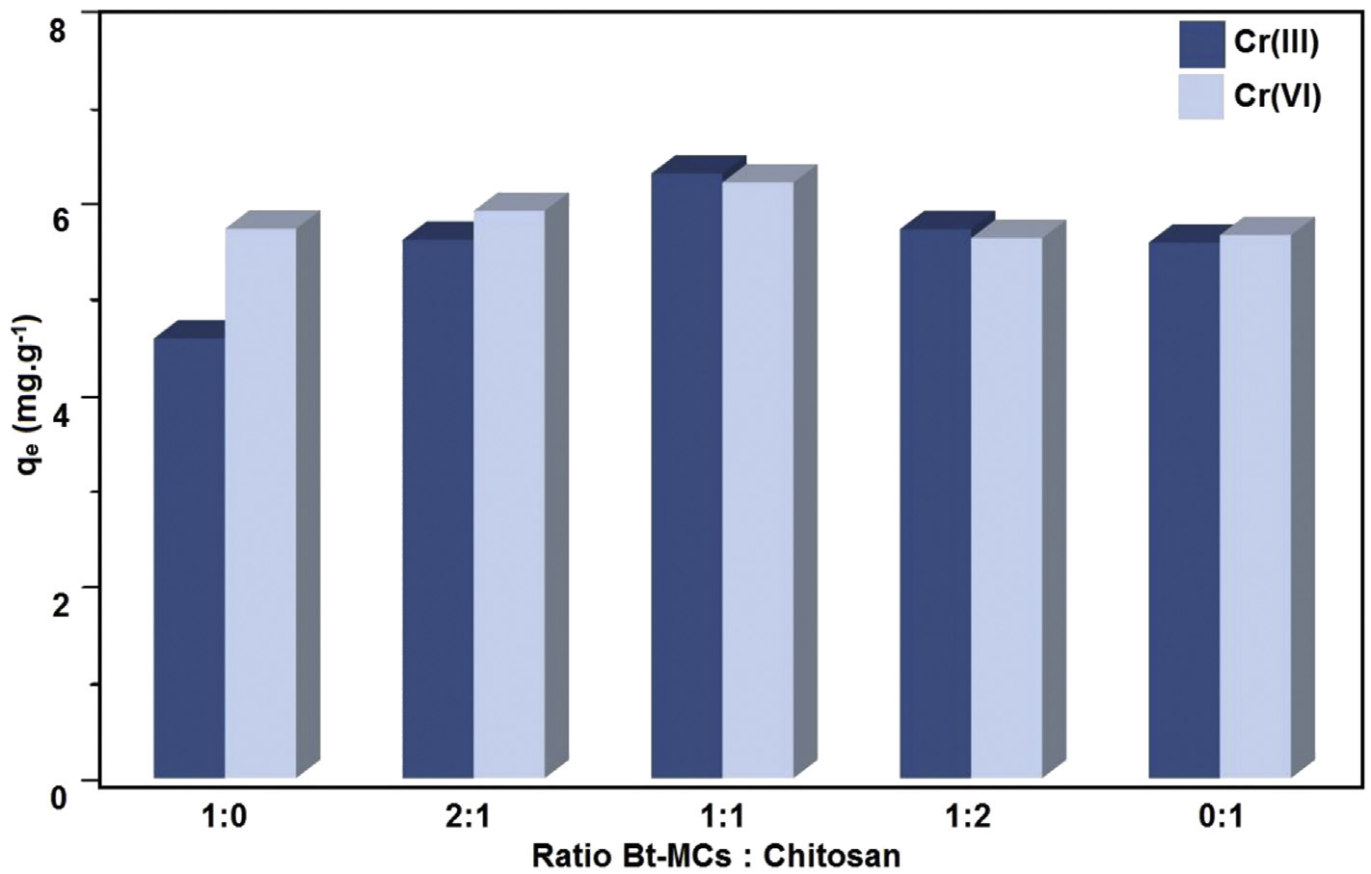
Synergistic effect between Bt-MCs and chitosan.

Using both CTAB and chitosan together as a modifier shows an increase of retention of Cr(III) and Cr(VI). This phenomenon is caused by the synergistic effect of the active group in chitosan and CTAB surfactant. Chitosan have –NH_2_ and –OH functional groups that has a good affinity for Cr(III) and can interact with anion species such as Cr(VI) depending on the pH of the solution [23]. CTAB as a quaternary ammonium groups can change the charge of bentonite surface from negative to positive, so it will be easy to interact with Cr(VI) [21]. In Figure 2, the synergistic effect show and concludes that Bt-MCCs have better performance for adsorption of Cr(III) and Cr(VI) than one modifier only and optimum ratio Bt-MCs and chitosan was 1:1. Effect of another parameter that has an effect on retention capacity in a batch system and physiochemistry characterization have been reported in the previous paper [21].

### 3.1 Optimization condition

**Figure 3:**
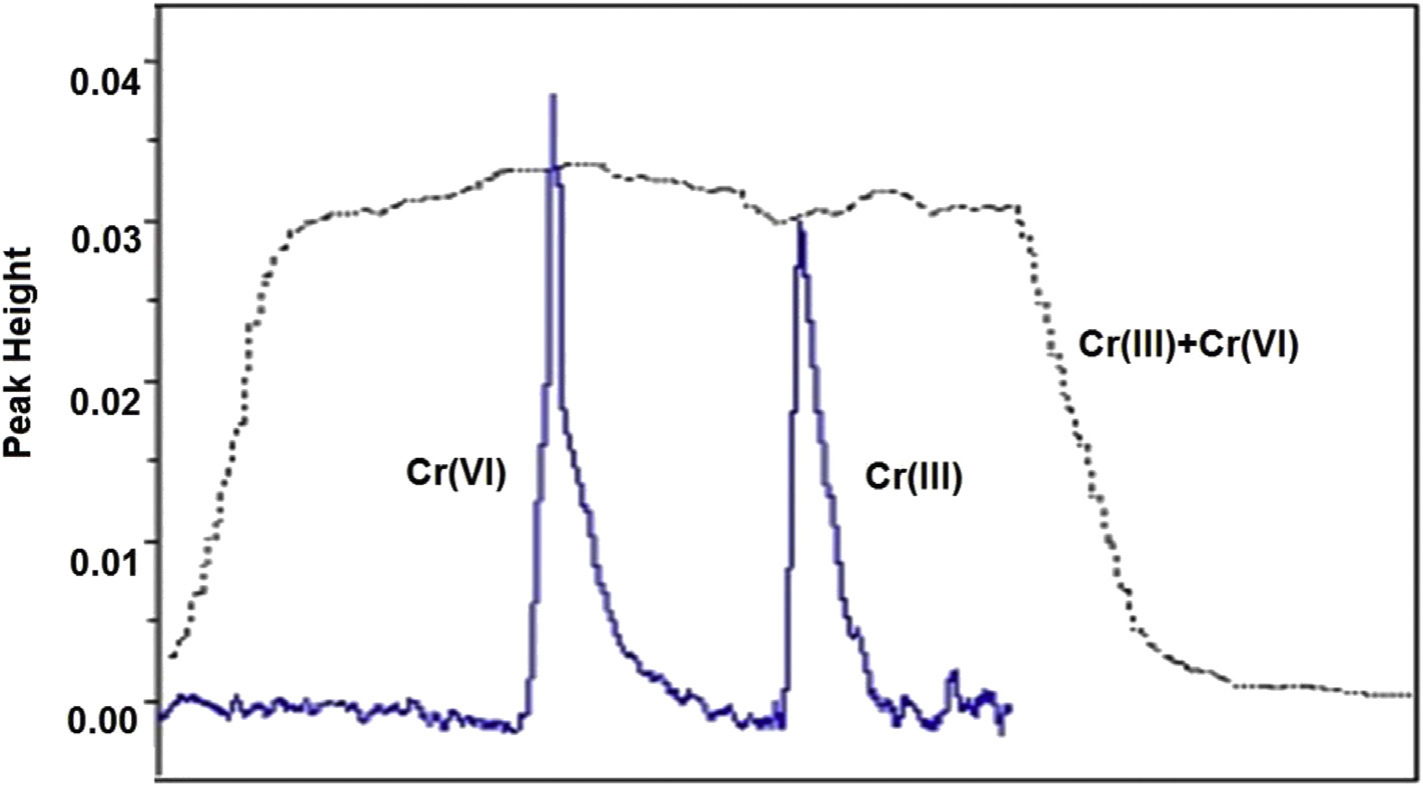
Comparison of Cr(III) and Cr(VI) peak height with and without preconcentration.

The Bt-MCCs mini column showed a good capability to preconcentrate Cr(III) and Cr(VI) ions. Figure 3 showed the comparison of the signal with and without preconcentration. A mixture of Cr(III) and Cr(VI) with the total concentration 2.0 mg.L^−1^ that was directly measured using AAS has the same peak height with a 1.0 mL of Cr(III) or Cr(VI) with each concentration of 0.1 mg.L^−1^ (equal to 0.1 μg Cr) that had preconcentration treatment. Generally, AAS only has the ability to analyze the total Cr in mg.L^−1^ unit and cannot differentiate Cr species. This fact showed the developed FIA-AAS system is effective to give information about the kind and concentration each of Cr(III) and Cr(VI) species. This results also showed possibility to use the Bt-MCCs mini column to preconcentrated Cr(III) and Cr(VI) ions with lower concentration.

**Figure 4:**
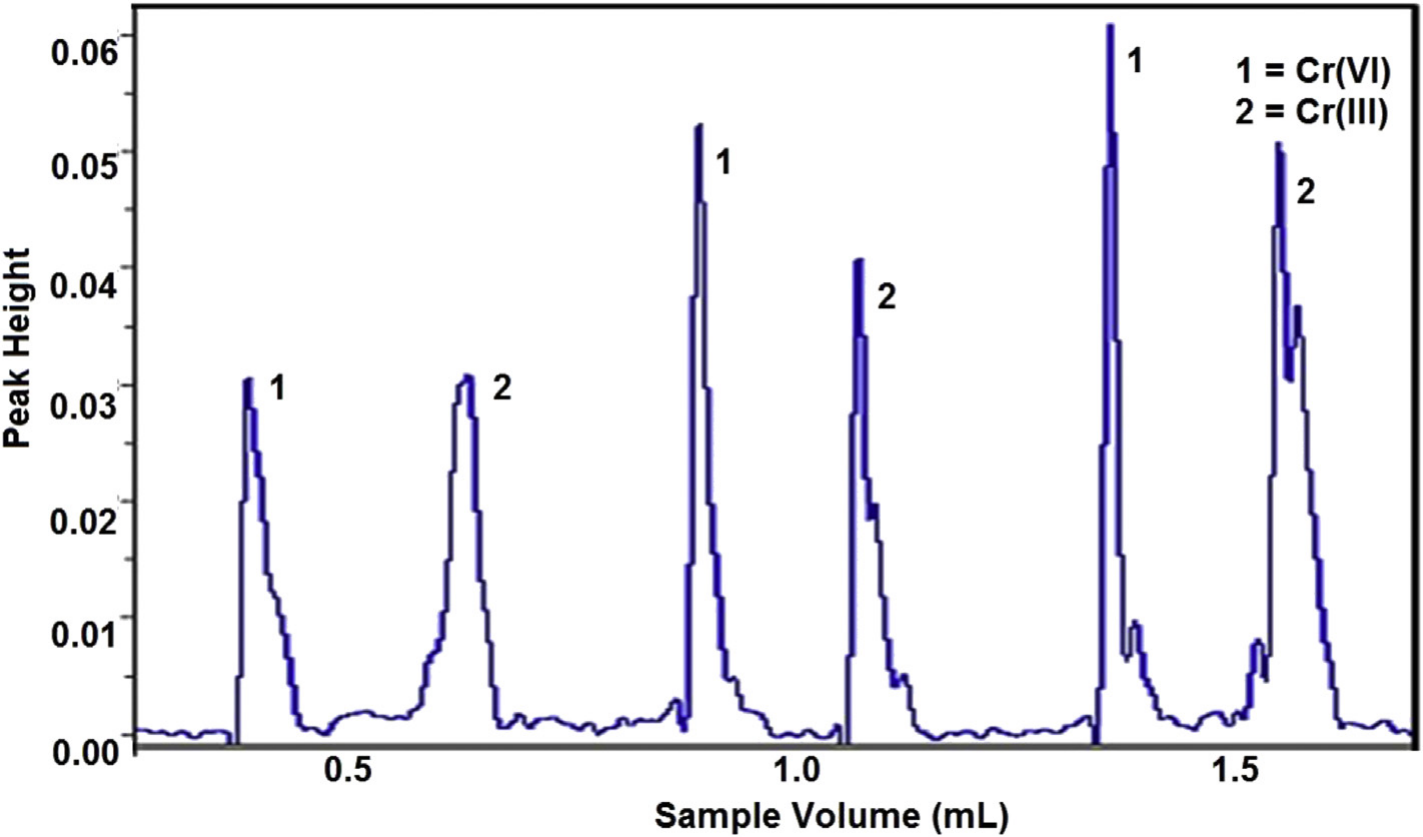
Effect of sample volume to fiagram of Cr(III) and Cr(VI).

We found the Bt-MCCs column not only has a good performance for low concentration sample, but also good for higher concentration. A mixture standard of Cr(III) and Cr(VI) with each concentration 0.5 mg.L^−1^ as sample and injected 0.5, 1.0 and 1.5 mL of it to get 0.5, 1.0, and 1.5 μg of Cr total. A fiagram in Figure 4 showed the increase of the sample volume with the same Cr species concentration that also increase the peak height of AAS signal. This result indicates that up until the concentration of analyte rises up to 15 times from the initial, the Bt-MCCs mini-column still has a good capability to absorb the Cr species. This results can explained from V_B_ capacity of column retention that is 3.13 and 2.25 mg g^−1^ for Cr(III) and Cr(VI), respectively [21]. For 0.2 g of Bt-MCCs mini column, it can retain up to 626.0 μg Cr(III) and 450.0 μg Cr(VI). That data means the dynamic retention capacity from the Bt-MCCs is still much bigger than the Cr injection from the sample, so both of volume and concentration sample can be enlarged to get bigger preconcentration factor.

**Figure 5:**
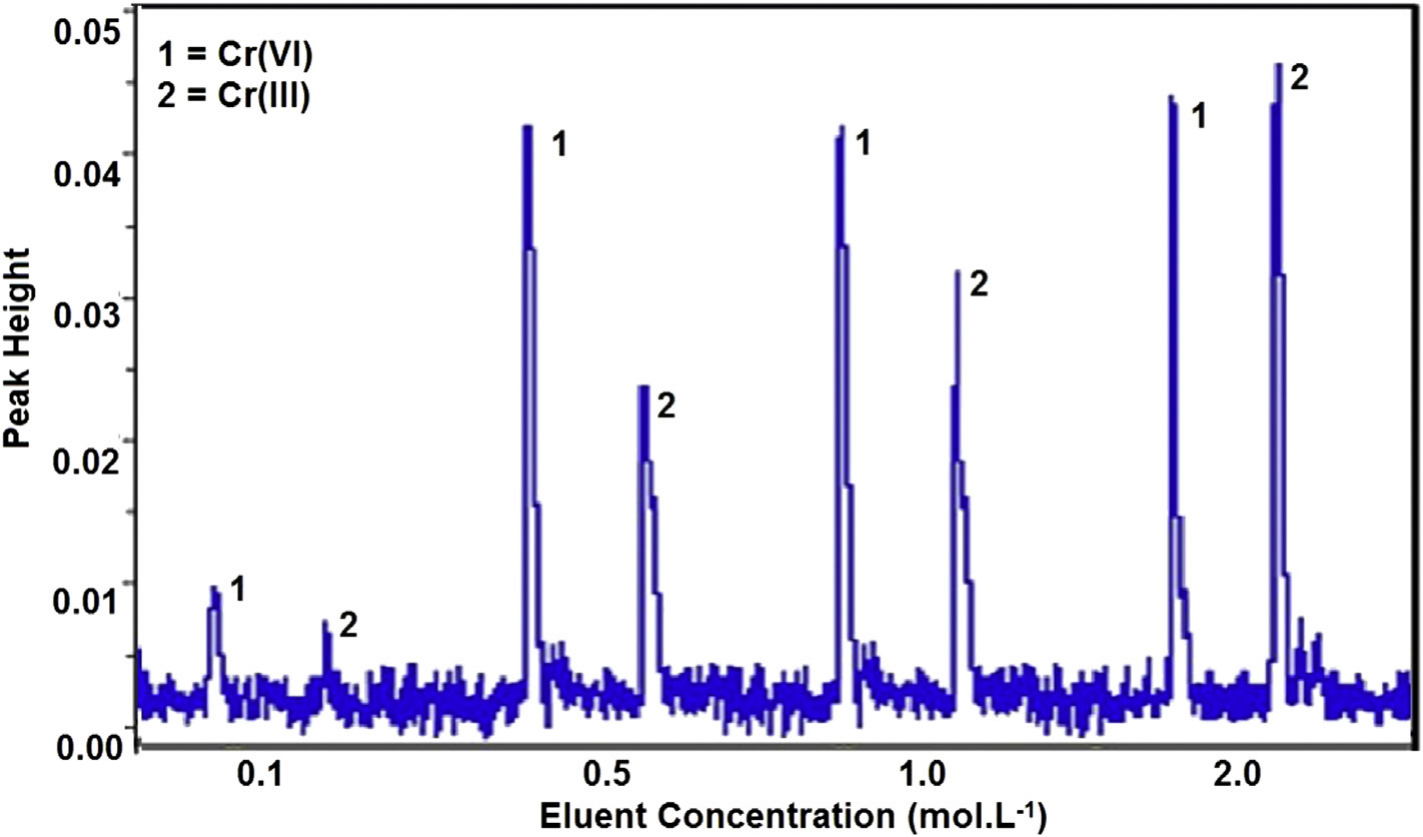
Effect of eluent concentration to fiagram of Cr(III) and Cr(VI).

**Figure 6:**
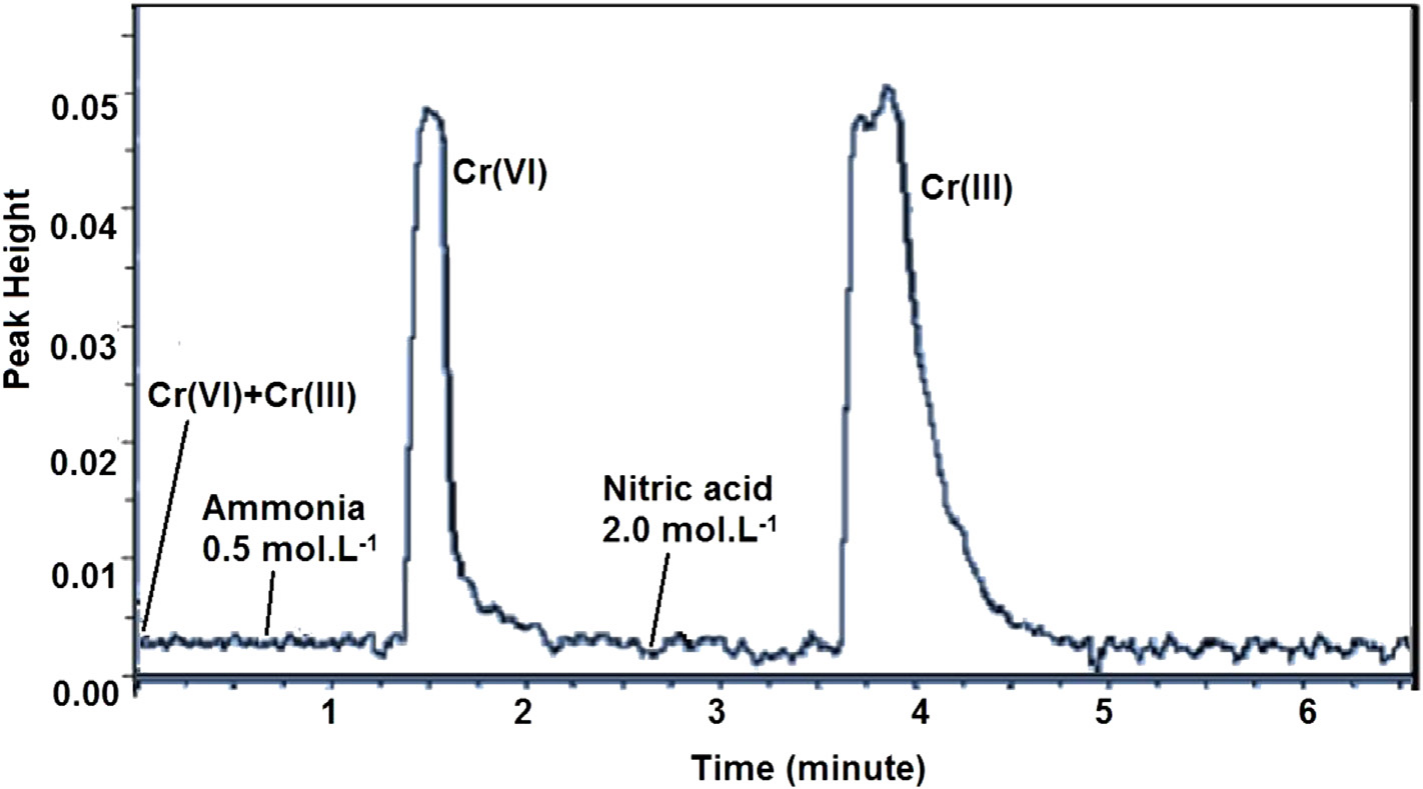
Fiagram of Cr(III) and Cr(VI) separation in optimum eluent concentration.

Elution is one of importance step after a retention process. A Cr(III) ion, which has a positive charge, can only interact with chitosan. A cation competitor is needed to weaken the interaction between Cr(III) and chitosan. We have used nitric acid to fulfill this condition. On the other hand, a Cr(VI) ion which has a negative charge will interacted with both of quaternary ammonium from CTAB and chitosan. With same logic, we have used ammonia to meet the condition. The order of elution will affect the quality of speciation. Half of Cr(VI) was also released when Cr(III) elute first, so we will elute Cr(VI) first. Eluent concentration between 0.1-2.0 mol.L^−1^ was used to evaluate effect of eluent concentration to quantitative signal of analyte. A 1.0 mL of Cr(III) and Cr(VI) mixture solution with each concentration 0.25 mg.L^−1^ was used for this analysis. A fiagram in Figure 5 showed that quantitative elution (>95%) was reached when minimal concentration 1.0 mol.L^−1^ for nitric acid to elute Cr(III). Unfortunately, concentration more than 2.0 mol.L^−1^ made the conditioning time longer and predicted that it will destroy the Bt-MCCs materials. A 0.5 mol.L^−1^ of ammonia solution already gave a maximum Cr(VI) elution. So for further analysis, nitric acid 2.0 mol.L^−1^ and ammonia 0.5 mol.L^−1^ were chosen. A fiagram of Cr(III) and Cr(VI) speciation in optimum concentration of eluent is presented in Figure 6.

**Figure 7:**
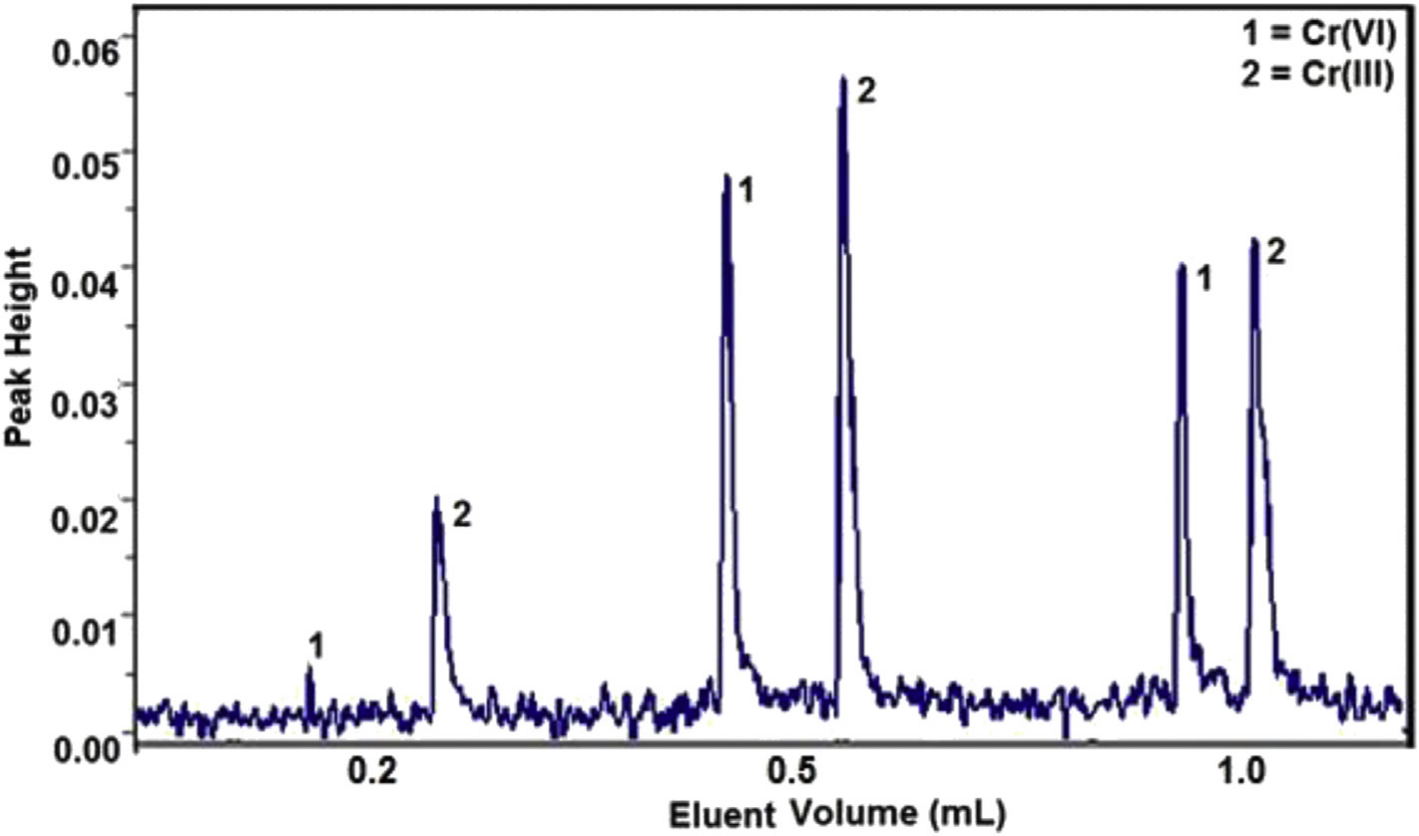
Effect of eluent volume to fiagram of Cr(III) and Cr(VI).

Ideal elution of sample can be reach if the analysis is fast, accurate, precision and consume less eluent [16]. Optimum eluent concentration was used to determine the eluent volume that produce optimum peak height. Eluent volume variation was controlled using a loop with volume 0.2, 0.5, and 1.0 mL. A fiagram in Figure 7 showed that both of nitric acid and ammonia give optimum elution when the volume of eluent 0.5 mL. Volume below 0.5 mL gave lower height peak because the elution is not maximal yet and bigger than 0.5 mL did not gave higher peak because dilution effect. Based on this fact, the next analysis would use a 0.5 mL of eluent.

### 3.2 Analytical performance

**Figure 8:**
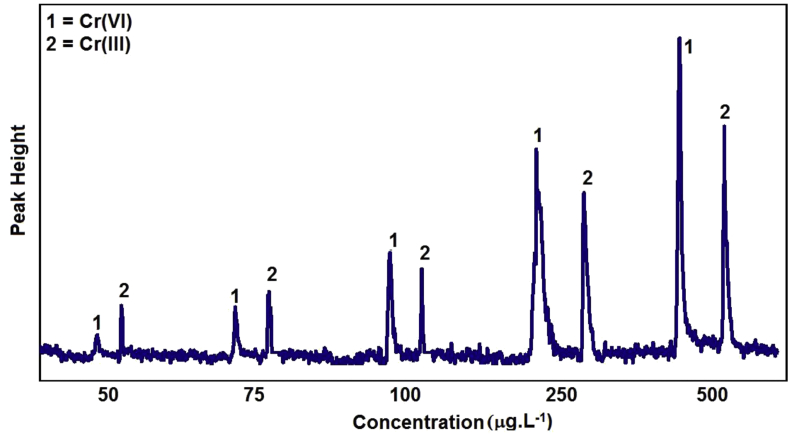
Fiagram profile of Cr(III) and Cr(VI) on various concentration in optimum condition.

To evaluate the analytical performance, we determined linearity, selectivity, repeatability, detection limit, enrichment factor, concentration efficiency and consumptive index. Linearity was determine based on linear regression of a certain range concentration of analyte. In this experiment, concentration between 50–500 μg.L^−1^ has a good relation between peak height (H) and analyte concentration ([Cr]) with dynamic range up to 12-fold. A fiagram profile of various concentration Cr species in optimum condition is presented in Figure 8. A linear regression plotting give correlation coefficient R^2^ = 0.9905 for Cr(III) and R^2^ = 0.9926 for Cr(IV). Both R^2^ value showed good linearity of this FIA-AAS system in the range of analyte concentration.

**Figure 9:**
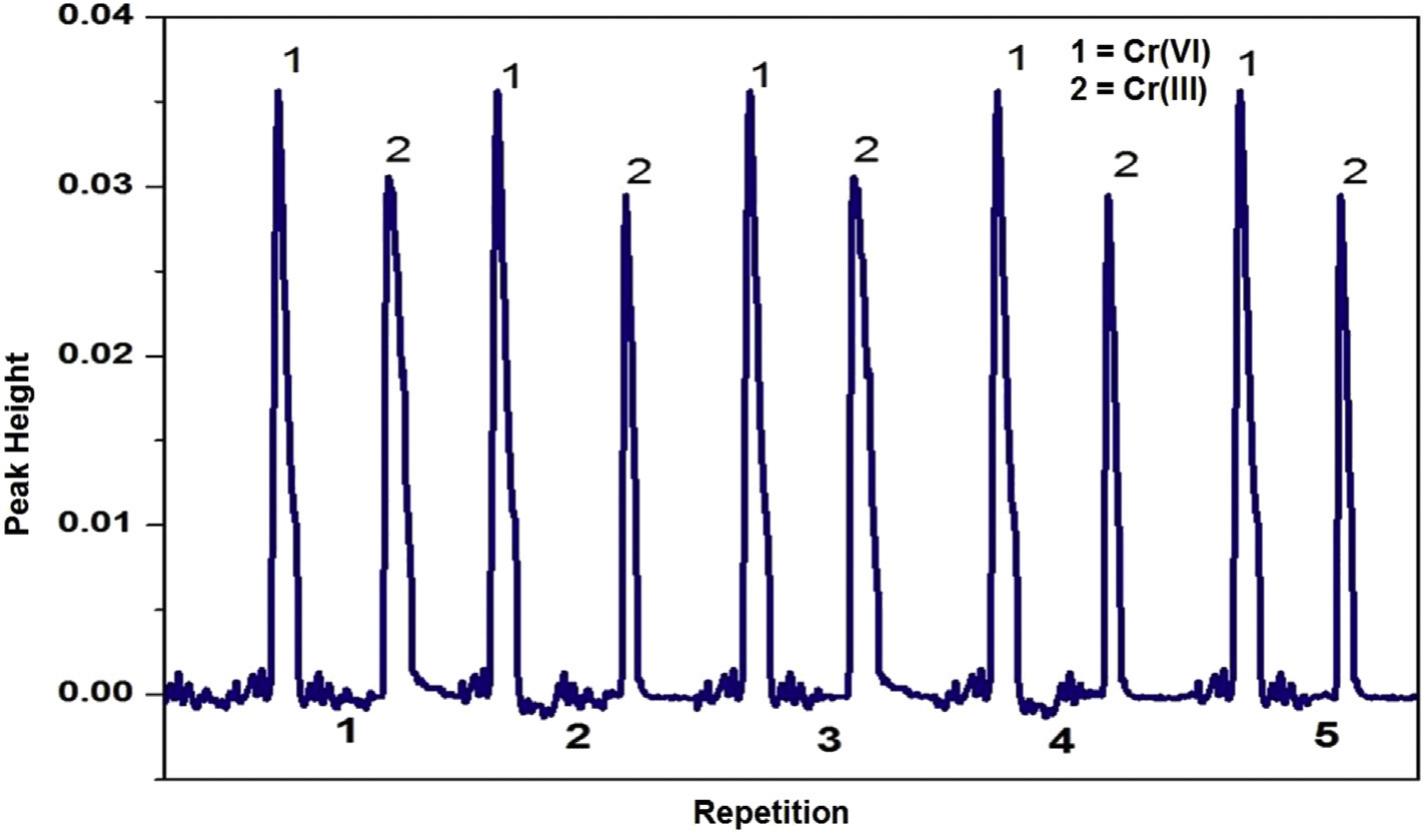
Fiagram profile of Cr(III) and Cr(VI) in optimum condition for five repetitions.

The % variance coefficient (%VC) is used to state quality of the analysis repeatability. Lower %VC means better precision. At optimum condition, fiagram like Figure 9 will be obtained. The %VC value of Cr(III) and Cr(VI) is 1.8 % and 0.6 %, respectively. The %VC value lower than 5.0 % indicates that the repeatability of developed analytical methods is very good. The Bt-MCCs mini column have been developed show good stability and performance for routine analysis.

The developed method has a limit detection consecutively 2.8 and 2.4 μg.L^−1^ for Cr(III) and Cr(VI) for 1 mL of sample based on limitation a S/N ratio = 3. The minimal mass detected (MMD) is 2.8 ng for Cr(III) and 2.4 ng for Cr(VI). Saygi et al reports they have a limit detection of 1.94 μg.L^−1^ as Cr(VI) for 25 mL minimal of sample [8]. It means that the MMD for this system is 48.5 ng. So, it can be concluded that this method has a better limit detection than another selective method that has been reported using AAS for ion detection. For too low concentration of Cr, this limit detection can be attained by enlarging the injection volume. But it needs to consider that more volume will make a longer analysis time and an effect to retention-elution process.

An enrichment factor (EF) describe ratio analyte concentration in sample before and after preconcentration. The EF calculated by comparing the slope of sample calibration curve with and without preconcentration. Table 1 showed a linear regression parameter for Cr(III) and Cr(VI) with and without preconcentration in equation

H = A + B[Cr], H = peak height; A = constant; B = slope; [Cr] = chromium concentration

**Table 1:** Regression parameter of Cr(III) and Cr(VI) with and without preconcentration.

Condition	Concentration range (μg.L^−1^)	Species	Parameter		
			A	B	R
Without preconcentration	1000–9000	Cr(III)	-0.0060	0.00003	0.9984
		Cr(VI)	-0.0028	0.00003	0.9964
With preconcentration	50–500	Cr(III)	-0.0037	0.0003	0.9905
		Cr(VI)	-0.0046	0.0003	0.9926

Calculation results showed preconcentration with the developed Bt-MCCs mini-column have EF 10-fold for 1 mL sample. This EF value can still improve by increasing the sample volume. Data in Table 1 also can use to calculate sensitivity. Sensitivity can be calculated using equation

S = 0.0044/k, S = sensitivity; k = slope of calibration curve

Calculation give the sensitivity are 14.7 μg.L^−1^ for both of Cr species.

The concentration efficiency (CE) value was determined by counting the analyte peaks. This method can be used in an hour. In this developed method, we can get two analyte peaks (Cr(III) and Cr(VI)) in 5 min, so CE for this method is 12 h^-1^. This value means that the developed method has a short analysis time and worth to use for routine analysis with high number of samples. Beside EF dan CE, consumptive index (CI) also can be used to evaluate the FIA performance. CI is a FIA parameter which is related to carrier and eluent volume. A FIA preconcentration supposedly have a small CI value to get an efficiency. In optimum condition, this developed system needs total volume 12.0 mL with detail 10 mL of carrier (2.0 mL min^−1^ x 5 min), 1.0 mL of sample, and 1.0 mL of eluent (2 eluent x 0.5 mL) to get 2 signal from different Cr species. The CI value maybe increase if the concentration sample very low, but EF value also increases too to compensate it without lose another analytical parameter.

### 3.3 Application for environmental sample

The sample that has been analyzed using this FIA-AAS system came from Cidurian river, West Java, Indonesia and taken in January 2017. The sample was filtered using filter paper to remove an undissolved particle and then the filtrate was analyzed using the optimum condition. To learn the matrix effect in analysis, we had used spike method and evaluated the percent recovery. Table 2 showed the percent recovery of river water sample for Cr(III) and Cr(VI). A higher percentage of recovery value indicates that the method has high accuracy and sample matrix does not give significant effect to the measurement.

**Table 2:** Concentration of Cr(III) and Cr(VI) in Cidurian river.

Sample	Cr concentration (μg.L^−1^)		Percent recovery (%)	Species
	Added	Found		
Cidurian river water	0	38.3 ± 1.8	98.8	Cr(III)
	100.0	136.7 ± 1.8		
	0	26.7 ± 0.8	100.7	Cr(VI)
	75.0	101.7 ± 0.8		

Table 2 also showed total concentration of chromium in river sample is 65.0 ± 2.6 μg.L^−1^. This value is too small for FAAS analysis because of inadequate sensitivity. Using the Bt-MCCs mini-column in FIA-AAS system solved that problem. Indonesian government regulation by Ministry of Environment and Forestry decree determine the threshold value for Cr total and Cr(VI) in water are 0.10 mg.L^−1^ and 0.05 mg.L^−1^ respectively. Sample analysis showed Cr species total is still below this threshold.

## 4 Conclusion

A Bt-MCCs mini-column can be prepared easily because, all of the chemical compounds are relatively easy to get and cheap. The column that has been used in FIA-AAS system shows a good analytical performance and can be applied to trace the analysis of Cr species. The column effectively function as a preconcentrator for low concentration Cr(III) and Cr(VI) in real sample matrix without losing the speciation performance. It is possible to decrease the concentration range of chromium species with increasing the volume sample injected to column. Increasing the sample volume of course effects the time to flow all the sample through the mini-column.

Speciation that was done using 0.5 mol.L^−1^ ammonia solution and 2.0 mol.L^−1^ nitric acid solution as specific eluent for each of chromium species. This eluent combination did not damage the column because the peak height has a good consistency after using it in many cycles. We suggest this technique for Cr(III) and Cr(VI) routine analysis in environmental sample because this technique have advantages like accurate, precise, and robust methods, modest equipment, short time for analysis, easily automated, low sample volume, easily to tandem with another equipment and low cost. An analytical performance summary is showed at Table 3.

**Table 3:** Summary of analytical parameter.

Parameter	Value	
	Cr(III)	Cr(VI)
Concentration range (μg.L^−1^)	50–500	50–500
Correlation coefficient	0.9926	0.9975
Sensitivity (μg.L^−1^)	14.7	14.7
Repeatability (%)	1.8	0.6
Limit detection (μg.L^−1^)	2.8	2.4
EF	10	10
CE (hour^−1^)	12	12
CI (mL)	12	12
Accuracy (%)	98.8	100.7

## Declarations

### Author contribution statement

Muhammad Bachri Amran: Conceived and designed the experiments; Analyzed and interpreted the data; Contributed reagents, materials, analysis tools or data; Wrote the paper.

Sitti Aminah: Conceived and designed the experiments; Performed the experiments; Analyzed and interpreted the data; Contributed reagents, materials, analysis tools or data; Wrote the paper.

Handajaya Rusli: Analyzed and interpreted the data; Wrote the paper.

Buchari, Buchari: Conceived and designed the experiments; Analyzed and interpreted the data.

### Funding statement

Sitti Aminah was supported by 10.13039/501100009509Kementerian Riset, Teknologi dan Pendidikan Tinggi (106/SP2H/LT/DRPM/IV/2017) Muhammad Bachri Amran was supported by P3MI Institut Teknologi Bandung (1275J/I1.C01/PL/2018).

### Competing interest statement

The authors declare no conflict of interest.

### Additional information

No additional information is available for this paper.
